# Supplementary material for: Broadly neutralizing antibodies against Omicron variants of SARS-CoV-2 derived from mRNA-lipid nanoparticle-immunized mice
Source: Heliyon. 2023 Apr 18;9(5):e15587. doi: 10.1016/j.heliyon.2023.e15587 (PMC10111857; doi:10.1016/j.heliyon.2023.e15587)
Supplement: Multimedia component 1 [file mmc1.pdf]

Supplemental Figure S1

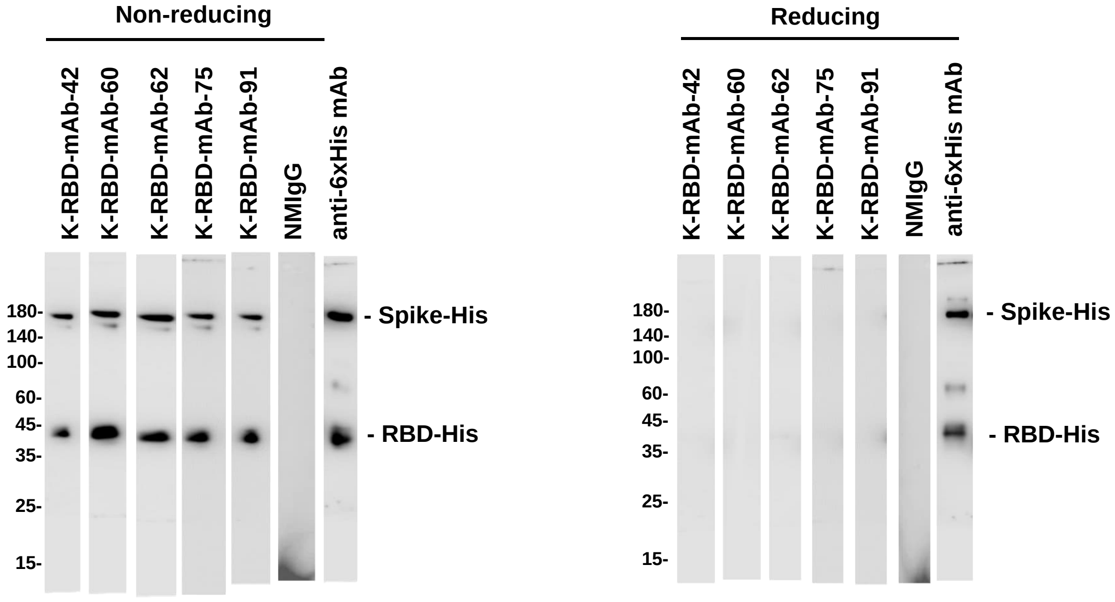

Figure S1. Western blotting Analysis of K-RBD-mAbs to Omicron BA.1 Spike and RBD protein. Five K-RBD-mAbs were used as primary antibodies for Western blotting. Each well was loaded recombinant BA.1 spike protein-His and RBD protein together. Anti-6×His mAb was used as a positive control. This data is supplementary of Figure 1D.
