# Supplementary material for: Broadly neutralizing antibodies against Omicron variants of SARS-CoV-2 derived from mRNA-lipid nanoparticle-immunized mice
Source: Heliyon. 2023 Apr 18;9(5):e15587. doi: 10.1016/j.heliyon.2023.e15587 (PMC10111857; doi:10.1016/j.heliyon.2023.e15587)
Supplement: Multimedia component 2 [file mmc2.pdf]

Supplemental Figure S2

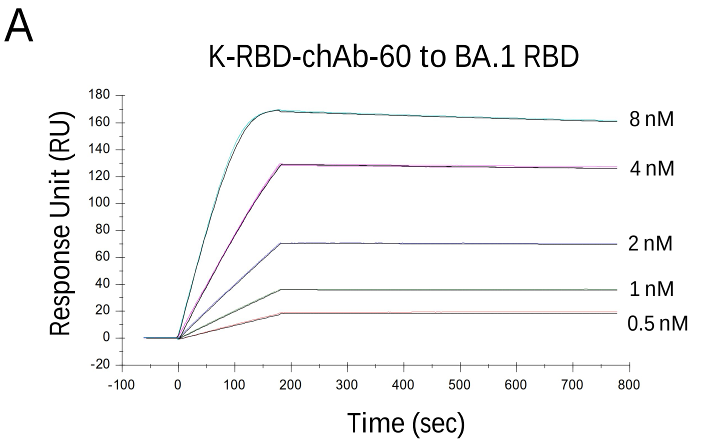

Report						
ka (1/Ms)	kd (1/s)	KD (M)	Rmax (RU)	tc	Chi2 (RU2)	U-value
7487213	8.58E-05	1.15E-11	168.6679	78748376	0.249089	3.137426

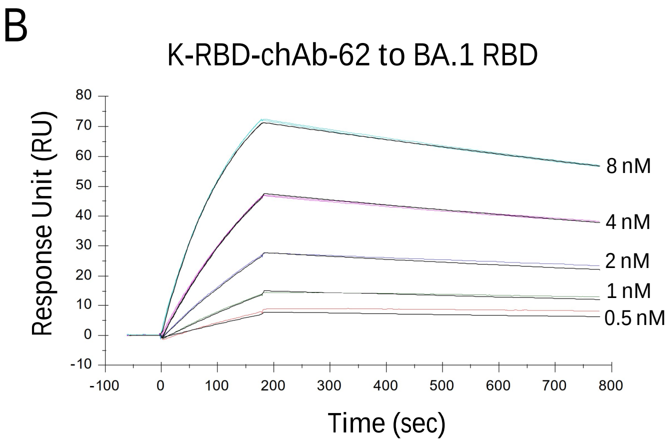

Report						
ka (1/Ms)	kd (1/s)	KD (M)	Rmax (RU)	tc	Chi2 (RU2)	U-value
974436.6	0.00038	3.9E-10	97.0835	1.22E+16	0.382877	0.845

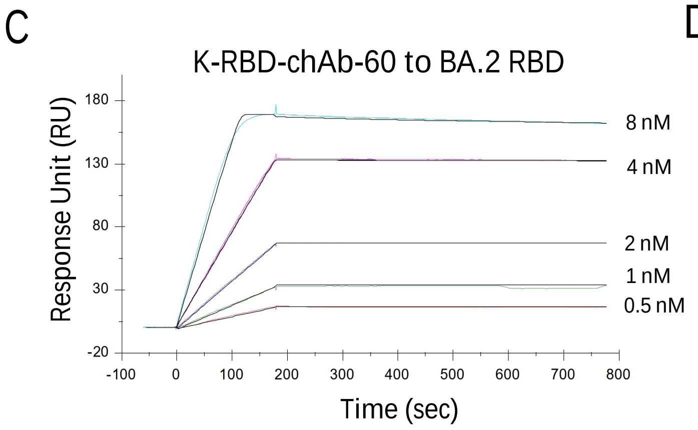

Report						
ka (1/Ms)	kd (1/s)	KD (M)	Rmax (RU)	tc	Chi2 (RU2)	U-value
66889035	9.63E-05	1.44E-12	167.1589	61498204	1.400581	11.64904

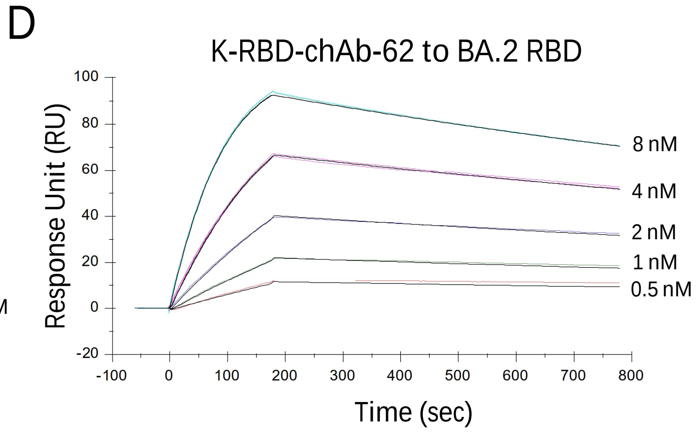

Report						
ka (1/Ms)	kd (1/s)	KD (M)	Rmax (RU)	tc	Chi2 (RU2)	U-value
1744674	0.000491	2.81E-10	106.4213	1.75E+08	0.296583	0.65

Figure S2. The binding kinetics of K-RBD-chAbs to recombinant Omicron BA.1 and BA.2 RBD protein. Analysis of the kinetics of the ligands (K-RBD-chAbs) to the analytes (BA.1 and BA.2 RBD protein) using BIAcore T200 system. Global fitted curves are shown. The KD values were calculated using a 1:1 binding model. Analyte concentration ranged 0.5 to 8 nM.
